# How Stressful Is Examining Children with Symptoms of Child Abuse?—Measurement of Stress Appraisal (SAM) in German Physicians with Key Expertise in Pediatrics

**DOI:** 10.3390/children9101578

**Published:** 2022-10-19

**Authors:** Louisa Thiekötter, Peter Schmidt, Marie-Léne Scheiderer, Heidrun Lioba Wunram, Michael Paulussen, Daniela Reis, Oliver Fricke

**Affiliations:** 1 Faculty of Health, Department of Medicine, Witten/Herdecke University, 58455 Witten, Germany; 2 General Pediatrics, Vestische Kinder- und Jugendklinik Datteln, 45711 Datteln, Germany; 3 Department of Special Care Dentistry, Witten/Herdecke University, 58455 Witten, Germany; 4 Department of Psychiatry, Psychosomatics and Psychotherapy for Children and Adolescents, Medical Faculty and University Hospital, University of Cologne, 50931 Cologne, Germany; 5 Department of Pediatrics, Medical Faculty and University Hospital, University of Cologne, 50931 Cologne, Germany; 6 Department of Child and Adolescent Psychiatry, Psychotherapy and Child Neurology, Gemeinschaftskrankenhaus Herdecke, 58313 Herdecke, Germany; 7 Department of Child and Adolescent Psychiatry and Psychotherapy, Klinikum Stuttgart, 70174 Stuttgart, Germany

**Keywords:** pediatricians, child protection, child abuse, stress appraisal measure, imagination study

## Abstract

Background: Pediatricians frequently feel uncertain about their ability to detect early symptoms of child abuse and how to respond in suspected cases. Aim: This study investigated the transactional stress model in German pediatricians who experienced imagination stories with a child protection scenario and another potentially stress-triggering scenario. Methods: A two-part survey was conducted online. Each part included a different imagination story and evaluation of the Stress Appraisal Measure (SAM), as well as questions on child protection, current problematics, and suggested remedies. In total, 96 pediatricians participated. The child abuse scenario was perceived as significantly more threatening and more stressful than a medical emergency. The pediatricians declared moderate familiarity with the Child Protection Guidelines and the Federal Child Protection Act and an average confidence in their application. The greatest perceived problems were communication difficulties with parents and youth welfare services. Suggested improvements were concrete procedural directives, more training programs, better interdisciplinary networks, and greater exchange among colleagues. Conclusions: To optimize their potential in the child protection system, pediatricians need to be better supported in coping with the identified stressors in child abuse scenarios.

## 1. Introduction

Pediatricians play a central role not only in the field of child healthcare, but also in the field of child protection [1,2]. An important aspect of the latter task is the early recognition of symptoms of abuse or endangerment for a child’s well-being [1,2,3].

In Germany, endangerment of child welfare (*Kindeswohlgefährdung*) is defined, in the terms of the Section 1666 I of the German Civil Code (*BGB*), as follows: “A child’s well-being is considered endangered if there is reasonable probability that continuation of a currently existing threat can significantly harm the child’s mental or bodily well-being” [4]. Signs of child maltreatment, abuse, or neglect are thus considered a credible indication that a child’s well-being is under threat [4]. Furthermore, the 11th revision of the International Statistical Classification of Diseases and related health problems (ICD-11) defines maltreatment as “Non-accidental acts of physical force, forced or coerced sexual acts, verbal or symbolic acts, or significant caregiving omissions that result in harm or have a reasonable potential for harm” [5].

Many countries, including Germany, offer pediatric examinations (in Germany, 10–12 during childhood and 1–2 during adolescence) that are widely accepted primary care [4,6,7]. Physicians with key expertise in pediatric or adolescent medicine are uniquely well-positioned to detect and respond to early signs of child abuse [1,4,8,9]. Nonetheless, in many cases child abuse remains undetected [2,10]. In 2018, for example, only 6.1% of the judicially reviewed cases of suspected child abuse in Germany had been reported by healthcare professionals [11]. Consequently, healthcare providers must either fail to identify child abuse or fail to initiate appropriate responses in many cases [11,12].

According to numbers from the Federal Statistical Office, the youth welfare services (*Jugendamt*) investigated 157,300 reported cases in which children were at a suspected risk of harm in Germany, in 2018 [13]. In 24,900 of these cases (16%), the threat to child welfare was identified as acute; in a further 25,500 (16%) cases, the threat was considered latent, and in an additional 34% of the cases, need for support and counselling was identified, although child well-being was not at risk [13]. In the 50,400 cases in which some level of risk had been identified, there had been symptoms of neglect in 60% of the cases, psychological abuse in 31%, physical maltreatment in 26%, and sexual abuse in 5% [13]. A high dark figure of unreported cases must, however, be assumed, and official numbers may frequently reflect only a small proportion of the actual cases [10,14,15,16]. A British study in this context thus found that the actual prevalence of child maltreatment was 7–17-fold higher than the official number of confirmed cases indicated [10].

In 2019, the Guidelines for Child Protection (*Kinderschutzleitlinie*) were issued in Germany to outline the responses and procedures pediatric professionals should follow in cases of suspected child abuse [4]. The aim of these guidelines, which offer advice on the diagnostic procedure in cases of maltreatment, abuse, and neglect, are to aid these professionals by increasing their confidence in how to handle suspected child abuse cases [4]. The Federal Child Protection Act (*Bundeskinderschutzgesetz*), which was passed in 2012, and the Act to Strengthen Children and Youth (*Kinder- und Jugendstärkungsgesetz*), which came into effect in 2021, have a similarly important function [4,17,18]. Both of these acts provide the legal basis for how to proceed in cases in which a child’s welfare is endangered [4,17,18]. They outline that whenever there is reasonable suspicion that a child’s welfare is in danger, pediatric health care professionals should first discuss their suspicion with the parents, or guardians, and children and suggest their referral to counselling and support services [4,18]. These acts also grant pediatricians some leeway in regard to patient confidentiality and allow them to discuss such cases with child protection experts, in anonymized form [4,18]. In cases where further action is deemed necessary to avert a risk of harm to the child, pediatricians are also permitted to contact to the youth welfare services; however, the child’s parents or guardians should be informed of this step beforehand [4,18]. Additionally, in 2021, the Act to Strengthen Children and Youth was passed, which mandates that cases in which there is imminent risk of harm to the children should be reported to the youth welfare services [4,17].

In view of the key role that pediatricians play in the early detection and prevention of child abuse [2,19], a relevant question in regard to child protection issues is in how far a suspicion of child abuse may trigger feelings of anxiety and diffidence, and thus elicit an acute stress reaction via cognitive appraisal and coping processes in these professionals. Due to the gravity of the diagnosis, the suspicion of child abuse needs to be well-weighed and supported by reasonable evidence [2]. Pediatricians may, hence, in light of these factors, conceivably experience feelings of tension and anxiety when they are confronted with potential child abuse situations. These feelings may possibly affect their response behavior.

An equally important aspect in this respect is to identify which factors induce stress in these situations; for example, whether pediatricians possess the necessary level of training to detect early signs of child harm or the procedural knowledge to follow the proper steps in the referral to the appropriate support or counselling services. In this context, an Italian study, for example, found that pediatricians and general practitioners had only scant knowledge on how to handle cases of child abuse and highlighted the need for proper training in this regard [20]. A study from Brittany, published in 2018, also illustrates that suspected cases of child abuse are frequently not reported by pediatricians [21]. The main reasons cited for this were lack of education on the topic, fear of misdiagnosing the situation, and lack of response from social services [21]. Additionally, in Germany, it is repeatedly pointed out that physicians working in the pediatric field experience a considerable degree of uncertainty in regard to their ability to detect early signs of child abuse, to respond appropriately, and to follow the proper procedure in suspected cases [22]. A study from Berlin thus showed that 73% of the surveyed pediatricians did not feel confident in their ability to detect early signs of child endangerment, and that 64% of the respondents wished for training programs on the topic [15]. A non-representative study conducted by the University of Ulm in Germany found similar results [11]. In that study, approximately 64–70% of the surveyed physicians and psychotherapists who worked with children responded that they were unsure of how to apply the Federal Child Protection Act, and only 15–25% were aware that they could report suspected cases to the youth welfare services, if the grounds for suspicion were reasonable [11]. These results support recent data published by Baker et al. [12]. The authors state that “trainings as a whole are not providing mandated reporters with comprehensive information about definitions, examples, and indicators of the major types of childhood maltreatment. In addition, the trainings contain only limited information to motivate reporters to see their role as part of a collective endeavor to protect children, and they are failing to adequately address reluctance about reporting” [12].

The studies and articles published to date on this theme mainly call attention to the existing uncertainties and problems experienced by pediatricians in child protection situations [15,21,23,24], or to the long-term emotional toll of treating maltreated or neglected children on these professionals, such as secondary traumatic stress or burnout [25,26]. None of the publications have so far, however, explicitly investigated to what extent such situations may also trigger acute stress in the treating physicians. To be able to accurately assess the degree of stress induced by child protection scenarios, the stress experienced in this type of situation needs to be compared to that from another potentially stress-triggering situation. The present study was, therefore, designed to assess and compare how pediatricians appraised two potentially stressful situations, using the SAM (stress appraisal measure). From a psychological perspective, the pediatricians’ cognitive appraisal and coping processes were of interest in this context. In terms of the transactional stress theory, the surveyed physicians were asked for that reason to assess two stress-triggering imaginary scenarios both with regard to their perceived level of threat, challenge, or importance, and with regard to the level of control they felt they had over the situation [27]. The main objective was to see if pediatricians in clinical practice experience a potential child abuse event as more threatening, challenging, significant, or less controllable—and thus as more stressful—than a medical pediatric emergency.

Another important aim was to identify stress-triggering factors in the context of child protection situations, as well as possible measures to reduce stress, e.g., more help or training courses. The study also investigated further aspects that might affect stress levels, like the influence of sociodemographic factors (age, sex), the amount of professional experience, or participation in professional training events.

## 2. Methods

***Two-part Survey:*** This questionnaire-based study is based on data collected in a two-part online survey of members of the German Association of Pediatric and Adolescent Care Specialists (BVKJ) (*Berufsverband der Kinder- und Jugendärzte e.V.*), which was conducted during two time periods. The data for the first part of the survey were collected in a time window from 23 February 2022 to 6 March 2022 (first survey part = t1); the data for the second part of the survey were collected in a time window from 8 March 2022 to 22 April 2022 (second survey part = t2). During the first part of the survey, there had been some respondents who had completed the questionnaire after the time window (t1) for this part had expired. These respondents were not invited to participate in the second part of the survey (t2) and were also not considered in the double participation evaluations. The BVKJ, which is an interest group formed by physicians with key expertise in pediatric and adolescent medicine in Germany that currently has approximately 12,000 members, strives to provide the best possible health services for children and adolescents and improve the necessary framework [28]. The BVKJ supported the data collection for both parts of the survey. Prior to the study, a positive vote for carrying out the survey was obtained from the board of the BVKJ. In addition, the study was approved by the Ethics Committee of the University of Witten/Herdecke (# S-38/2022) on 16 April 2022.

An invitation to participate in the study was extended to interested pediatricians through the BVKJ association’s newsletter (distributed only to members), which included a link to the study’s online participation pages. The survey was conducted using the freely available SoSciSurvey tool (www.soscisurvey.de), an online platform which functions in accordance with European data protection norms. After agreeing to the privacy policy, interested pediatricians could participate in the surveys. After the first survey period, participants were invited to participate in the second survey. Participants who failed to respond to the second survey were sent a maximum of two reminders. Both the invitation and the reminders were sent directly to the participants via their e-mail addresses. For the data evaluation, every participant was then given a pseudonym (serial number) to ensure anonymity, so the given responses in the surveys could no longer be linked to the e-mail addresses. The data from both surveys were compiled on a dedicated “response” server.

In the first survey (t1), the questionnaire comprised five questions, an imagination story describing a fictive case scenario, and 28 items from the validated German version of the Stress Appraisal Measure (SAM) [27,29]. In the second survey (t2), the questionnaire comprised seven questions, an imagination story, and 28 items from the validated German SAM. The SAM is a psychometric instrument to capture stress perceptions rooted in transactional cognitive processes that mediate coping behaviors in response to acute stressors [27,29]. For the purposes of this study, the participants were, therefore, exposed to imagination stories with fictive case scenarios. In t1, the participants were confronted with the scenario of a physical medical emergency (febrile child with a seizure), as described in Figure S1: Imagination story 1; in t2, the participants were confronted with a child protection scenario (suspicion of child maltreatment), as described in Figure S2: Imagination Story 2. During a pre-test that was run prior to the actual study, both imagination stories had been assessed for their clinical pertinence and realism at two German pediatric clinics (Vestische Kinder- und Jugendklinik Datteln, Gemeinschaftskrankenhaus Herdecke). In the study, the stress induced by the fictive scenarios in the imagination stories, as well as the cognitive appraisal and coping processes related to these scenarios, were then assessed using the SAM [27,29]. In the first part of the survey (t1), personal data, such as age (in categories), sex, place (Federal State), years of professional experience (in categories), and field of activity (clinic, practice, public health service, etc.) were collected in addition to the SAM scores. These data were needed for verification and evaluation purposes. By collecting only categorical responses to these questions, the anonymity of the participants was ensured, and their identities remained obscure. In conclusion to the second part of the survey (t2), the participants were also asked to respond to questions related to aspects of child protection, such as participation in professional training programs and degree of confidence in applying the Federal Child Protection Act and the Child Protection Guidelines, as well as to perceived difficulties and suggested improvements. The purpose of these questions was to identify areas with the potential need for targeted action to improve the child protection system.

***Measurement of cognitive appraisal and evaluation processes with the SAM:*** The central goal of this study was to assess the cognitive appraisal and coping processes elicited by two stress-triggering situations and to compare the two situations. This was accomplished using the German version of the SAM, validated by Delahaye et al. [27]. The SAM, which was originally developed by Peacock and Wong, is a questionnaire-based instrument that allows a multidimensional approach to assessing a current stress-triggering event [29]. This psychometric instrument is based on a stress model that was conceptualized by Lazarus and Folkman, in which an individual’s reaction to a stressor elicits two types of cognitive appraisal, primary and secondary appraisal [30]. While primary appraisal involves the fundamental evaluation of a situation (dangerous, pleasant, irrelevant), secondary appraisal focusses on the extent of situational control an individual has, i.e., the ability to control an event or to cope with its demands [30]. According to Lazarus and Folkman, stress is experienced whenever the demands of a situation that was appraised as being dangerous in the first appraisal step exceed the individual’s perceived resources or ability to control the situation in the second step [27,30]. Cognitive appraisal of a situation is thus a fundamental element of stress [30]. The SAM instrument comprises seven scales with a total of 28 items, with three scales each pertaining to the dimensions of the primary and secondary appraisal of stress [27,29]. In addition, there is a scale to index perceived overall stressfulness [27,29]. The assessed dimensions of primary appraisal are threat, challenge, and centrality, while those of secondary appraisal are dimensions of perceived control (controllable-by-self; controllable-by-others; uncontrollable [27,29]. Each of the items was rated on a five-point Likert scale (1 = not at all; 5 = a great amount) [27,29].

***Data analysis:*** The data were analyzed using descriptive statistics (frequencies; mean values). First, the data for all participants from t1 who had completed the survey were analyzed. These data were later compared with those from t2 (*n* = 96; t1 and t2). Only the data from the 96 participants who had completed both parts of the survey were considered in the further analyses.

Unweighted indexes (t1 and t2) were calculated by taking the mean value of the four items in each SAM subscale. Cronbach’s *α*-coefficient was then determined to assess the internal consistency of the items in the seven subscales. It is, however, difficult to assess overall reliability with this approach [31]. Alpha-coefficient values <0.80 are considered low, values between 0.80 and 0.90 are considered average, and values >0.90 are considered high [32]. To compare the participants’ responses for both parts of the survey, the mean values for each of the SAM subscales for t1 and t2 were compared by performing a *t*-test for dependent samples. To counteract alpha-error accumulation due to multiple testing, the predetermined significance level of *p* = 0.05 was reduced to *p* = 0.05/7 = 0.01, in correspondence with the separate tests for the seven SAM subscales [33]. Results with p-values lower than *α* = 0.01 were considered significant. In a further step, Cohen’s d was calculated to determine the effect size. Values > 0.80 show a large effect, values > 0.50 a medium effect, and values > 0.20 a small effect [34].

Subsequently, for the data from the second survey, which dealt with endangerment of child welfare, Levene’s test was first performed to test the homogeneity of the variance before a univariate, multifactorial analysis of variance was conducted [35]. These steps were undertaken so that determinants that could have an effect on the overall perceived level of stress, and thus on the results of the survey, could be considered and compared. Determinants that were taken into account in this context were sociodemographic factors, such as age and gender, professional experience, and the number of training courses taken in the field of child protection. The significance level for the variance analysis was placed at *α* = 0.05. The result was considered significant for *p*-values < 0.05.

## 3. Results

***Pre-test:*** Before beginning the study, the realism and relevance to day-to-day clinical practice of the two imagination stories was confirmed by 29 pediatricians. Suggestions for re-phrasing and minor amendments to the stories were adopted.

***Sociodemographic Data and Child Protection Aspects:*** For survey t1, 143 completed questionnaires were returned. Most of the respondents were female (*n* = 105; 73.4%), aged between 40 and 49 years (*n* = 49; 34.3%), and had 20–29 years of professional experience (*n* = 56; 39.2%). Moreover, 121 of the respondents worked in a pediatric practice (84.6%), and 77 lived in the Federal States of Nordrhein-Westfalen, Bayern, or Baden-Württemberg (53.9%).

As this was a comparative study, the further analyses were conducted only for the *n* = 96 participants for which values for the relevant variables were available from both survey parts. As can be seen in Table 1, in the longitudinal view, more women (*n* = 67; 69.8%) than men (*n* = 29; 30.2%) participated in the study. The largest age group was that of 40–49 years old (*n* = 34; 35.4%). The majority of respondents worked in pediatric practices (*n* = 86; 89.6%) and had between 20 and 30 years of pediatric experience (*n* = 37; 38.5%). Most of the respondents (*n* = 58; 60.4%) came from in the Federal States of Nordrhein-Westfalen, Bayern, and Baden-Württemberg, and 42.7% (*n* = 41) of the respondents stated that they had attended one or two training events in the field of child protection over the last ten years.

The second part survey (t2) also contained questions with regard to the participants’ levels of confidence in applying the Federal Child Protection Act and the Child Protection Guidelines and their perception of problems and suggestions for improved child protection measures in the pediatric field. The responses to these questions showed that the participating pediatricians had a moderate level of familiarity (*M* = 3.22; *SD* ± 0.81) with the contents of the Federal Child Protection Act and the Child Protection Guidelines, and an average level of confidence (*M* = 3.18; *SD* ± 0.77) in how to apply these in practice. The most frequent responses given by the participants with regard to the greatest difficulties they encountered in situations in which they suspected a risk of harm to a child’s welfare are given in Table 2. Table 2 also lists the most frequent responses with regard to what kind of help or training opportunities pediatricians would like to see offered. With regard to child protection issues, 65.6% (*n* = 63) of the surveyed pediatricians wished for greater support or more training opportunities.

### Measurement of Cognitive Appraisal and Coping Processes with the SAM Questionnaire

***Psychometric characteristics of the SAM subscales:*** The Cronbach’s *α-*coefficients for the seven SAM subscales, or dimensions, were as follows: for “threat” (*αt*1 = 0.76; *αt*2 = 0.72), for “centrality” (*αt*1 = 0.88; *αt*2 = 0.85), for “controllable-by-self” (*αt*1 = 0.92; *αt*2 = 0.86), for “controllable-by-others” (*αt*1 = 0.83; *αt*2 = 0.88), for “uncontrollable” (*αt*1 = 0.63; *αt*2 = 0.68), for “challenge” (*αt*1 = 0.24; *αt*2 = 0.16), and for “overall stressfulness” (*αt*1 = 0.80; *αt*2 = 0.77). The *α-*coefficients for “centrality,” “controllable-by-self,” and “controllable-by-others” thus indicated average reliability, and the coefficients for “overall stressfulness” indicated a low-to-average reliability. In contrast, low reliabilities were found for the subscales “threat,” “uncontrollable-by-anyone,” and “challenge.” The reliability for the subscale “challenge” deviated most strongly from the reliabilities for the other subscales.

***Perception of stress and cognitive appraisal SAM:***Table 3 compares the mean values and standard deviations for the subscales of the SAM instrument for surveys t1 and t2. The results of the *t*-test for dependent samples, based on the comparison of the mean values for the SAM subscales (for t1 and t2), were as follows: For the subscale “threat,” the difference between mean values (*M* = −0.36; *SD* = 0.83) was significant (*t*(95) = −4.26; *p* < 0.001), with a large effect size (*Cohen’s d* = 0.83). Significant differences (*t*(95) = −2.97; *p* = 0.002) between mean values (*M* = −0.23; *SD* = 0.76) were also found for the subscale “centrality,” with a medium effect size (*Cohen’s d* = 0.76). The mean value differences for the secondary appraisal subscales “controllable-by-self” (*M* = 0.37; *SD* = 0.71; *t*(95) = 5.12; *p* < 0.001) and “uncontrollable” (*M* = −0.44; *SD* = 0.64; *t*(95) = −6.65; *p* < 0.001) were also significant, with medium effect sizes both for the subscales “controllable-by-self” (*Cohen’s d* = 0.71) and “uncontrollable” (*Cohen’s d* = 0.64). In contrast, the mean value differences for the subscales “challenge” (*M* = 0.05; *SD* = 0.62; *t*(95) = 0.74; *p* = 0.23) and “controllable-by-others” (*M* = −0.08; *SD* = 0.87; *t*(95) = −0.94; *p* = 0.18) were found to be insignificant. On the other hand, the mean value differences between t1 and t2 for the subscale “overall stressfulness” were, again, significant (*M* = −0.24; *SD* = 0.89; *t*(95) = −2.63; *p* = 0.005), with a large effect size (*Cohen’s d* = 0.89).

***Other factors affecting the perception of stress:*** The results of the univariate, multifactorial analysis of variance, which was performed to investigate the possible influence of age, gender, professional experience, and number of attended training events on the perception of overall stress triggered by the child-abuse imagination story in survey t2 were as follows: The variances of the dependent variables in the different categories, which were determined by the Levene test, as a prerequisite for this analysis, were homogenous because the test was not significant (*p* = 0.09).

The greater the number of training events that had been attended within the last 10 years, the smaller the mean values for perceived stress, as measured in the subscale “overall stressfulness,” were: no training events: *M* = 4.13, *SD* = 0.58; 1–2 training events: *M* = 3.45, *SD* = 0.16; 3–4 training events: *M* = 3.37, *SD* = 0.18; ≥5 training events: *M* = 3.34, *SD* = 0.22. However, the difference between the number of attended training events over the last ten years and overall stress levels were not statistically significant (*F*(3.56) = 1.64, *p* = 0.19).

The effect of years of professional experience in pediatric medicine on the perceived level of stress was (*M* = 3.31; *SD* = 0.41) in the category of <5 years of experience. This measure increased in the category of 5–9 years of experience (*M* = 3.75; *SD* = 0.33), and then fell again with increasing years of experience (10–19 years: *M* = 3.60, *SD* = 0.19; 20–29 years: *M* = 3.34, *SD* = 0.18; ≥30 years: *M* = 3.25, *SD* = 0.21). Additionally, here, there was no statistically significant difference in mean values (*F*(4.56) = 1.21, *p* = 0.32).

No significant mean value differences or significant effects on the measured stressfulness were found for gender (*F*(1.56) = 0.01, *p* = 0.94) or for the different age categories (*F*(3.56) = 1.20, *p* = 0.32) between survey t1 and survey t2.

## 4. Discussion

Although the majority of the surveyed pediatricians felt moderately familiar with the contents (*M* = 3.22; *SD* = ±0.81) of the Child Protection Guidelines and the Federal Child Protection Act, and averagely confident about how to implement these in practice (*M* = 3.18; *SD* = ±0.77), the participants’ cognitive appraisal and perceptions of stress differed for the two imagination stories. The imagination story with the child protection scenario was, on average, rated as more threatening, more central, and more stressful than the medical emergency scenario. Similarly, the level of control-by-self was seen as lower in the child protection scenario than in the medical emergency. Our finding that child protection scenarios trigger high levels of stress in pediatricians are corroborated by a Canadian study, which reports a high prevalence of stress and burn out among multidisciplinary hospital-based physicians working in the field of child protection [26].

In the evaluation of our results for other factors influencing stress, the univariate analysis of variance for gender, age, professional experience, and participation in training events on the topic of child protection did not show a significant effect of any of these factors on the measured stress level. In this respect, our results are comparable with those of a British study that investigated the development of acute stress reactions and post-traumatic stress disorder in junior pediatric doctors, following their involvement in the death of a child [36]. This study also did not find a significant correlation between age, gender, years of work, experience on pediatric ICU wards, education on child death, or other pertinent training courses and the development of stress reactions [36].

While average reliabilities were found for almost all of the SAM-subscales, the low internal consistency found for the subscale “challenge” (αt1 = 0.24; αt2 = 0.16) can be seen as a limitation of this study. On the other hand, Delahaye et al. also found an α-coefficient of only 0.57 for the “challenge” subscale in their study [27]. In addition, a different study that used the German version of the SAM questionnaire reported an α-coefficient value of 0.33 for this subscale, and the authors noted that item 19 had correlated negatively with some of the other items for this subscale [37].

One of the greatest problems the surveyed participants perceived in responding to situations in which they suspected the endangerment of child welfare was that of having to communicate their suspicion to the child’s or adolescent’s parents or guardians. This step was considered particularly difficult due to the challenge of preserving a relationship of trust while still protecting the child’s welfare. Similar concerns were reported in a study that interviewed specialized child-abuse physicians, who reported that the relationship between doctor and patients or their parents, or guardians, in child-abuse settings was not comparable with the relationship known to occur in normal doctor–patient relationships [23]. The child-abuse physicians reported how difficult it was to strike a balance between good cooperation with the parents on the one hand and child safety on the other [23].

Another problem named by the participants in our study was the quality of communication with the youth welfare services. The participants were particularly troubled by the circumstance that the youth welfare services were often either difficult to reach or failed to provide feedback on reported cases. The lack of response by child and youth welfare services in cases of child endangerment has also been frequently pinpointed as a problem in international studies [21,24,38]. In Germany, the Act to Strengthen Children and Youth, which came into force in 2021, has now made it mandatory for the youth welfare services to respond to child professionals who report reasonable suspicions that a child’s well-being could be at risk [4,17]. Future studies will be needed to evaluate how this mandate is implemented in practice. Additionally, a medical child protection hotline has been installed in Germany to help address this issue. This telephone counselling service, which is available in all of the federal states, offers around-the-clock telephone counselling for professionals working in the field of child and youth protection [39].

In addition to communication problems with parents, guardians, youth welfare services, and other interdisciplinary professionals, many participants also reported difficulties in recognizing and diagnosing suspicious cases, particularly, if the symptoms were ambiguous. Other studies covering this aspect have reported similar findings to the effect that pediatricians often feel they lack the confidence to decide whether signs that appear suspicious are in fact indications of child abuse [21,23,24]. This fear of misdiagnosing child abuse, in combination with the possibly weighty consequences of the diagnosis, were found to trigger stress in many physicians and diffidence in regard to whether or not to act on the suspicion [15,23,24]. Likewise, 73% of pediatricians and pediatric psychiatrists surveyed in a study in Berlin said they found it difficult to recognize the early signs of child endangerment [15].

Although the majority of the participants in our study felt moderately familiar with the content and averagely confident about the practical application of the Child Protection Guidelines and the Federal Child Protection Act, which both outline how to handle child protection cases in Germany within the legal framework [4,18], many subsequently admitted to still having concerns about the legal aspects of such situations. This result is in accord with the responses given by pediatricians and pediatric psychiatrists in the study from Berlin: Although 68% of these practitioners had said they were familiar with the legal regulations, only approximately half of the respondents were aware that they had the right to consult with a child protection specialist if they anonymized the case [15]. In the study from Berlin, 70% of the participants had also expressed a wish for legally mandatory reporting of suspected cases of child endangerment to improve the legal situation for pediatricians in Germany [15].

To be able to identify possible measures to reduce stress and to improve the confidence of pediatricians when they are confronted with child protection issues, the study participants had been asked to relate their wishes and suggestions for improvements in this context. Frequent suggestions were further training opportunities on the topic, as well as concrete directives on how to proceed in such cases in practice. Despite the introduction of the Child Protection Guidelines, which were compiled to provide practical recommendations [4], the surveyed pediatricians saw the need for more help in this regard. This need for more decisive procedural directives has also been identified in numerous other national and international studies [15,20,21,40]. Furthermore, the pediatricians wished for better regional and interdisciplinary networks, and, especially, concrete addresses and contact persons to whom they could turn to in urgent cases when support measures and other interventions to safeguard a child’s wellbeing needed to be initiated as quickly as possible.

The detailed, multifaceted responses in our study—not only with regard to improvements in the field, e.g., more help or training events, but also with regard to the difficulties pediatricians encounter in child endangerment situations—illustrate the pertinence of this topic for primary care pediatricians. The results of this study, which identify some of the stressors pediatricians encounter in child abuse settings and underscore their need for more decisive procedural directives and better training opportunities, will hopefully, in the long run, contribute to an easier implementation of child protection measures in daily practice and help increase the effectiveness of the child protection system. Emotional response and communication skills are, therefore, important aspects that should be included in pediatric training schedules which can be ascertained despite the limited sample size of our study collective.

Despite improvements and advances in this regard in Germany in recent years (e.g., Child Protection Guidelines, Federal Child Protection Act, and the Act to Strengthen Children and Youth) [4,17,18,39], this study is, nonetheless, a call for better measures to help pediatricians cope with the identified stressors in child abuse scenarios and thus optimize their potential in strengthening the child protection system.

## Figures and Tables

**Table 1:** Characteristics of the study participants (members of the BVKJ).

Background Characteristics of the Study Participants	*n* = 96	
***Gender***		
Women (*n*/percentages)	67	69.8%
Men (*n*/percentages)	29	30.2%
***Age*** ***(in age groups*** ***)***		
**30–39 (*n*/percentages)**	**17**	**17.7%**
30–39 years/men (*n*/percentages)	3	3.1%
30–39 years/women (*n*/percentages)	14	14.6%
**40–49 years (*n*/percentages)**	**34**	**35.4%**
40–49 years/men (*n*/percentages)	6	6.3%
40–49 years/women (*n*/percentages)	28	29.1%
**50–59 years (*n*/percentages)**	**27**	**28.1%**
50–59 years/men (*n*/percentages)	10	10.4%
50–59 years/women (*n*/percentages)	17	17.7%
**≥60 years (*n*/percentages)**	**18**	**18.8%**
≥60 years/men (*n*/percentages)	10	10.4%
≥60 years/women (*n*/percentages)	8	8.4%
***Current field of activity***		
Practice	86	89.6%
Clinic	4	4.2%
Public health Service	1	1%
Currently no medical work	1	1%
Others	4	4.2%
***Years of professional experience in pediatrics***		
<5 years	4	4.2%
5–9 years	7	7.3%
10–20 years	29	30.2%
20–30 years	37	38.5%
<30 years	19	19.8%
***How many training events in the field of child protection have you attended in the past 10 years?***		
None	2	2.1%
1–2	41	42.7%
3–4	34	35.4%
≥5	18	18.8%
Missing value	1	1%

Abbreviations: German Association of Pediatric and Adolescent Care Specialists (BVKJ) (Berufsverband der Kinder- und Jugendärzte e.V.).

**Table 2:** Confidence of the pediatricians, as well as difficulties and potential remedies in the area of child protection.

	1 and 2	3	4 and 5	*M* (*SD*)
*Pediatricians’ level of confidence* *				
How confident do you feel about the content of the Child Protection Guidelines and the Federal Child Protection Act? (*n* = 1 missing value) *	18.8% (18)	41.7% (40)	38.5% (37)	3.22 (±0.81)
How confident do you feel about the practical implementation of the Child Protection Guidelines and the Federal Child Protection Act? (*n* = 1 missing value) *	17.7% (17)	46.9% (45)	34.4% (33)	3.18 (±0.77)
***Questions with open-ended answers***	***frequently mentioned answers***			
Where do you personally see the greatest difficulties when there is suspicion that a child is endangered?	communication with parents (address suspicion, relationship of trust vs. assistance) communication with youth welfare services (e.g., accessibility and feedback) and interdisciplinary communication recognition of suspicious cases and diagnosis legal basis regional and interdisciplinary networks (e.g., concrete contact persons) feedback from youth welfare services after reporting a suspicious case practical recommendations for action regular training courses and handouts, exchange with colleagues			
Would you like more help or further training opportunities in the area of child protection and, if so, what would help you the most?				

* The answers were given on a scale in which 1 = not at all, 2 = a bit, 3 = medium, 4 = quite a lot, 5 = fully; *M* = Mean value; *SD* = Standard deviation.

**Table 3:** Mean values and standard deviations for survey t1 and survey t2 SAM subscales.

	1. Survey t1		2. Survey t2	
Subscale SAM	Mean	*SD*	Mean	*SD*
**Threat SAM** *	2.15	0.66	2.51	0.74
**Centrality SAM** *	2.38	0.87	2.61	0.82
**Controllable-by-self SAM** *	4.21	0.64	3.84	0.68
**Controllable-by-others SAM** *	3.89	0.76	3.97	0.80
**Uncontrollable SAM** *	1.26	0.44	1.70	0.59
**Challenge SAM** *	3.16	0.52	3.11	0.53
**Stressfulness SAM** *	3.26	0.77	3.50	0.77

* (five-point Likert scale (1 = not at all; 5 = a great amount); Explanation: *SD* = standard deviation, SAM = Stress Appraisal Measure.

## Data Availability

The data presented in this study are available on request from the corresponding author. The data are not publicly available due to privacy reasons.

## Supplementary Materials

The following supporting information can be downloaded at: https://www.mdpi.com/article/10.3390/children9101578/s1, Figure S1: Imagination story 1, Figure S2: Imagination story 2.
