# Supplementary material for: How Stressful Is Examining Children with Symptoms of Child Abuse?—Measurement of Stress Appraisal (SAM) in German Physicians with Key Expertise in Pediatrics
Source: Children (Basel). 2022 Oct 19;9(10):1578. doi: 10.3390/children9101578 (PMC9600667; doi:10.3390/children9101578)
Supplement: Supplementary file 1 [file children-09-01578-s001.zip › children-1958268-supplementary.pdf]

children

MDPI

Supplementary Materials

You will now be presented with a short imagination story. Please read it carefully and then answer the following questions.

Please imagine the following scenario:

On a Friday morning, a mother enters your practice with her five-year-old daughter, Emma. You already know them from previous visits to your practice and you remember Emma as a cheerful and generally well-developed child. After you have seen two other patients, you ask the five-year-old patient and her mother to come into your practice. Emma has had a fever of up to 40.2°C since the previous day and is generally very weak and tired. Before you start taking the medical history and do the physical examination, you notice that Emma is suddenly staring into space and no longer responds to you. Shortly thereafter, the girl overstretches her head, stretches her legs for a few seconds, and then enters a state in which her arms and legs twitch rhythmically, without regaining consciousness.

Figure S1. Imagination story 1.

You will now be presented with a short imagination story. Please read it carefully and then answer the following questions.

Please imagine the following scenario:

On a Friday morning, a mother enters your practice with her two-year-old daughter, Hanna. This is their first visit to your practice, so you do not know the family yet. Today, a preventive medical checkup is scheduled. While you are talking to the mother before the checkup, you find out about the preventive medical check-ups that have been performed so far. You notice that the mother and her daughter have already been to two different paediatricians, and you also notice that the U5 checkup was skipped. When asked, the mother briefly reports that she did not feel in good hands with her previous doctors. At the time of the U5 checkup, she did not have a paediatrician for Hanna, so the checkup was not done. During your subsequent physical examination of Hanna, you notice several hematomas of different ages, especially on her buttocks and lower back. You also notice that these bruises run in a pattern of parallel bands. Hanna also has isolated bruises on both of her shins. You ask her mother for an explanation for the numerous hematomas. She reports that Hanna is an active child who moves around a lot and bumps herself often. Her mother cannot, however, name specific situations in which the bruises occurred.

Figure S2. Imagination story 2.
